# Dining Tables Divided by a Border: The Effect of Socio-Political Scenarios on Local Ecological Knowledge of Romanians Living in Ukrainian and Romanian Bukovina

**DOI:** 10.3390/foods10010126

**Published:** 2021-01-08

**Authors:** Nataliya Stryamets, Giulia Mattalia, Andrea Pieroni, Ihor Khomyn, Renata Sõukand

**Affiliations:** 1 Department of Environmental Sciences, Informatics and Statistics, Ca’ Foscari University of Venice, Via Torino 155, 30172 Venice, Italy; giulia.mattalia@unive.it (G.M.); renata.soukand@unive.it (R.S.); 2 University of Gastronomic Sciences, Piazza Vittorio Emanuele 9, 12042 Pollenzo, Bra, Italy; a.pieroni@unisg.it; 3 Medical Analysis Department, Tishk International University, Erbil 44001, Kurdistan Region, Iraq; 4 Nature Reserve “Roztochya”, Sitchovuh Strilciv 7, 81070 Ivano-Frankove, Ukraine; igor.homyn@ukr.net

**Keywords:** cultural landscapes, marginal rural areas, non-wood forest products, rural livelihoods, wild plants, wild food

## Abstract

Local cuisine is an important reservoir of local ecological knowledge shaped by a variety of socio-cultural, economic, and ecological factors. The aim was to document and compare the current use of wild and semi-cultivated plant food taxa by Romanians living in Romania and Ukraine. These two groups share similar ecological conditions and historically belonged to the same province, but were divided in the 1940s by the creation of a state border. We conducted 60 semi-structured interviews with rural residents. The contemporary use of 46 taxa (plus 5 cultivated taxa with uncommon uses), belonging to 20 families, for food consumption were recorded. Romanians in Romanian Bukovina used 27 taxa belonging to 15 families, while in Ukraine they used 40 taxa belonging to 18 families. Jams, sarmale, homemade beer, and the homemade alcoholic drink “socată” are used more by Romanians in Southern Bukovina, while tea, soups, and birch sap are used more in Northern Bukovina. We discuss the strong influence of socio-political scenarios on the use of wild food plants. Cross-ethnic marriages, as well as markets and women’s networks, i.e., “neighbors do so”, may have had a great impact on changes in wild food use. In addition, rapid changes in lifestyle (open work market and social migration) are other explanations for the abandonment of wild edible plants.

## 1. Introduction

The way people prepare their food is an important expression of local culture that is also shaped by the surrounding environment and historical background [1]. Indeed, different cultures often develop different recipes and the use of different ingredients. The underlying ecological knowledge is in fact shaped by a number of factors including socio-cultural aspects, like religion [2], language [3], politics including governance systems [4,5,6], and economic features [7]. Such knowledge is not static, but rather dynamic as it evolves over time according to changes in the abovementioned factors. For instance, a change in lifestyle (influenced by economic shifts) was found to change the perceptions and the use of wild food plants [8,9,10] by changing motivations such as the desire for quality food and the pleasure of gathering wild plants [11], and also as a consequence of the disappearance of an agrarian lifestyle [12]. Wild food plants play an essential role in traditional cuisines across Europe [13], not to mention their economic value, especially in marginal rural areas in countries undergoing economic and governance transition [14]. The study of wild food plants in multicultural contexts has only been partially researched among migrant communities (e.g., [15]) and among coexistent ethnic groups (e.g., [16]), yet this is of vital importance not only to understand past food habit trajectories [17], but also to foresee possible future scenarios. To explore the trajectories of wild food plant consumption, Bukovina is an ideal case study due to its multiculturalism and complex historical background. This region, now split between Romania and Ukraine, is a hotspot of cultural diversity as it was home to several minority groups, including Jewish, Polish, German, and Hungarian people [18]. During the Austro–Hungarian Empire, Bukovina was a land of immigration with people coming from different contexts (e.g., [19,20]). Such complex human ecology, along with the peculiar landscape consisting of the Carpathian Mountains as well as hilly and plains areas, resulted in a varied and rich food heritage. However, more recent political events have also resulted in consistent emigration from both Romanian and Ukrainian Bukovina to other European contexts as well as urban centers of the respective countries. Such a phenomenon may have contributed to changes in the current consumption of wild food plants, yet only one recent investigation has demonstrated the influence of globalization on the use of wild plants in terms of social mobility [21]. In Ukraine, only a handful of publications have addressed current or past uses of wild food plants (e.g., Western Ukraine [7,22,23], Bukovina [4,5,6], and Northern Ukraine [24], in addition to a historical analysis of the use of plants [25] and a questionnaire among specialists working in the country [26]). In Romania, there have been ethnobotanical studies on the use of wild food ([24] and reference therein) [27], focusing on different ethnic groups (e.g., Hungarians [28,29], Italians [30], Polish [31], Tatars and Romanians [32], and Hutsuls and Ukrainians [4,33]). Yet, there have been no studies on the use of wild plants for food by the Romanian diaspora or Romanian ethnic groups living in other countries. 

Within this multifaceted and intriguing context, the Ukrainian–Romanian border area of Bukovina has been settled by, among others, Romanians, Ukrainians, and Hutsuls (a Ukrainian ethnic minority living predominantly in Hutsulshyna and mountainous areas of Bukovina [34,35,36]), which have shared a complex history and socio-economic scenario, but different governance systems [4]. A few studies on the influence of the border in Bukovina with regard to the use of wild plants have been published [4,5,6,37]. However, the rich biocultural diversity of border areas due to different cultures, ethnos, religions, rituals and multiplex geopolitical and socio-economic conditions in a relatively small space [38] requires more research [39]. This is even more important in the current era of globalization and the homogenization of cultures, languages and natural resource use [40,41,42], as biocultural diversity is dynamic, involving both ecological and cultural relationships, changing the use of wild plants due to a variety of external factors [42].

During the COVID-19 crisis, with the various logistical problems and unstable situation with regard to the food supply and food security, wild food has developed a new important role as a safety-net. Therefore, study of the contemporary use of wild plants for culinary purposes, and the driving forces increasing or decreasing their use, is a timely endeavor. In this new reality, knowledge of the plants that surround us could be vital, especially in marginal rural areas. In the past, the use of wild foods has played various roles, and those roles are changing [11,12].

Within this multicultural border, we propose here a cross-border study to document and compare the current and past uses of wild and semi-cultivated plant food taxa by Romanians that live in similar ecological conditions and historically belonged to the same province, but were divided between two countries (Romania and Ukraine) in the 1940s. Moreover, we aimed to compare the uses of wild taxa among Romanians living on both sides of the border with other ethnic groups (e.g., Hutsuls). 

Our hypothesis is that the different political scenarios of the two countries (Romania and Ukraine) have led to different socio-economic phenomena (e.g., emigration, seasonal work abroad, intense contact and cohabitation with neighbors through cross-ethnic marriage, and the influence of other food cultures (via markets and mass media)) which have affected the use of wild plants for culinary purposes in the historically unified region of Bukovina. It would be logical to expect that Romanians living on both sides of the border use similar wild food plants as they share the same language and, since 2015, can freely move across the border. Yet, even with an “untrained eye” one can see that Romanians living under the same environmental conditions, in the same historical province, but on opposite sides of the border, have different wild food plant uses. However, the question remains: to what extent do the living conditions of the country and the social interconnections that exist there influence local cuisine and wild food use?

## 2. Materials and Methods

### 2.1. Study Areas

Bukovina province has long been inhabited by Ukrainians and Romanians. It is believed that the name derives from the fact that the region was covered by natural beech (*Fagus sylvatica*) forest: in Ukrainian “бyк”—buk means beech tree (the word has a German root) and “Bukovina” means the beech land [43].

A complex history under different governance systems, e.g., the Turkish Empire, Moldavian Kingdom, Austro–Hungarian Empire (1774–1918), Kingdom of Romania (1918–1940), and then division into Southern and Northern Bukovina between Romania and the Soviet Union, respectively, have influenced population structure and distinctive natural resource use [18]. Therefore, in the study region, the population predominantly consisted of Hutsuls in the mountainous part of Bukovina [35] as well as Romanians and Ukrainians (the latter inhabiting the lowlands of Northern Bukovina). The multicultural population of Bukovina was formed under the Austro–Hungarian law freeing local inhabitants from taxes for 30 years, which led to the growth of the population, from 80 thousand in 1800 to 850 thousand at the beginning of the 20th century, via migrants from other overpopulated regions of the Austro–Hungarian Empire, such as Ukrainians, Germans, Polish people, Jewish people, Hungarians, Roma people, etc. [18,34]. Bukovina was characterized by ethno-political stability, as there were no cross-ethnic conflicts [18]. The division of Bukovina between Romania and the Soviet Union in 1940 led to changes in the multicultural population structure, especially in Northern Bukovina. Soviet governors initiated depopulation and forcibly sent Romanians, Germans and Polish people away [44]. In Southern Bukovina, Jewish people were deported and Hungarians and Germans moved out [45]. Rich Ukrainians were sent to Siberia and, later, the Romanians who stayed in Northern Bukovina (in the territory of the Soviet Union) did not have the opportunity to migrate to Romania. There are historical data that many Romanians were killed while trying to escape from the Soviet Union [44,45].

**Figure 1:**
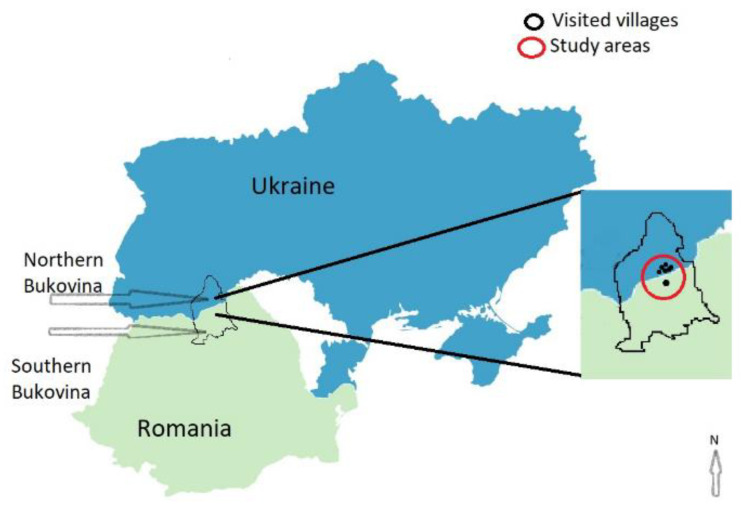
Study areas in Ukraine (Northern Bukovina) and Romania (Southern Bukovina).

The study was conducted in a lowland area of Northern Bukovina, Ukraine and the uplands of Southern Bukovina, Romania (Figure 1).

After Romania joined the European Union (2007) the economic situation improved and crossing the border to purchase consumer goods became popular [46]. In 2015, the Romanian and Ukrainian governments signed an agreement on “small cross-border movement” [47]. This allows residents of Romania and Ukraine who permanently live in the 35–50 km border zone to cross the border without obtaining a visa. People have to apply for a card, which is issued once every two years, and then they can cross into the other country, but not outside the 35–50 km small movement zone. There is a list of villages on both sides that are included in this agreement, and our study villages appear on this list. This agreement allows Romanians living in Ukraine to freely travel to visit their relatives and vice versa [46]. This also includes economic benefits for both areas as residents now commute and buy cheaper or better groceries on both sides of the border. In June 2018, Ukraine signed an agreement with the European Union establishing no-visa entry for Ukrainian residents possessing biometric passports to member states of the Schengen area.

**Figure 2:**
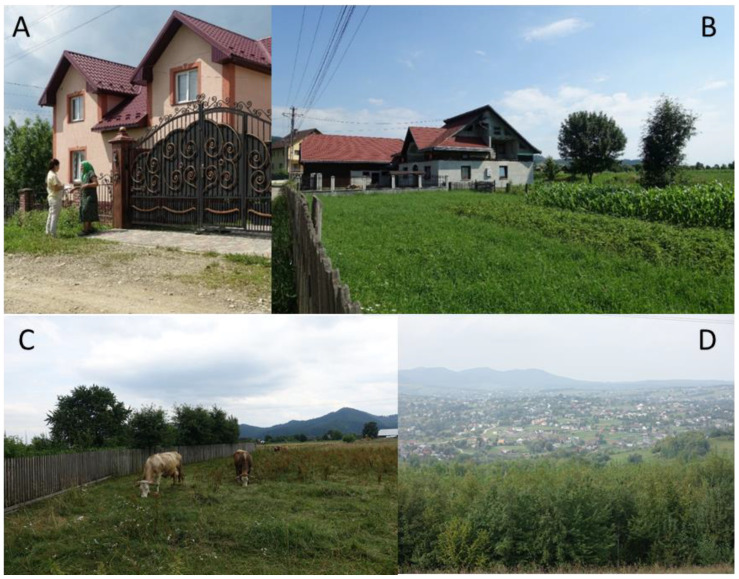
The local landscape in Northern (**A**,**D**) and Southern (**B**,**C**) Bukovina. Photos by: (**A**) S. Nagachevskyi, summer 2018; (**B**–**D**) N. Stryamets, summer 2019.

**Figure 3:**
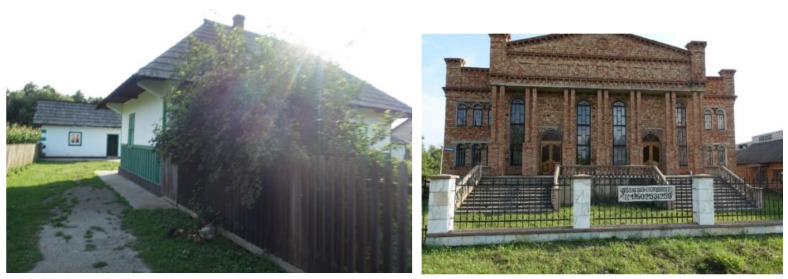
A Romanian house with wooden roof (**left**) and a newly built house in the same village for which the advertisement says “For sale” (**right**), in Northern Bukovina. Photos by N. Stryamets, August 2018.

Our participatory observation in Northern Bukovina in 2018–2019 revealed a large number of cars with foreign plates (from Romania, Great Britain, Germany, and others). As was explained by locals, to officially register a car in Ukraine costs a lot of money, sometimes more than the price of the car, so locals use foreign license plates. This also helps to travel abroad, because for a Ukrainian registered car you need to buy insurance. Economic development in the area is seen in brand new houses built close to old ones with wooden roofs (see Figure 2 and Figure 3). Even though it is still Northern Bukovina, the Hutsul area of Putyla [4] is much poorer with a worse infrastructure compared with the region of Storozenetskyi and Gluboka where Romanians and Ukrainians live. 

Every household engaged in small-scale family farming, including both animal breeding (mainly cows, chickens, ducks, and goats, and rarely sheep) and vegetable gardens for home consumption (mainly corn, cabbage, potatoes, onions, garlic, beetroot, beans, and carrots, as well as many other crops).

### 2.2. Northern Bukovina, Ukraine and Southern Bukovina, Romania

Northern Bukovina corresponds to the southern–eastern part of Chernivci region (except Hotynchshyna). The landscape consists of agricultural fields and some beech forests (Table 1). The Glybotskyi and Storozenetskyi region is situated in the foothills of the Carpathian Mountains and is located in the Prut-Sirets interfluve. The highest peak is 475 m a.s.l.

Romanians live in villages close to the Romanian border, in the regions of Glybozkij, Gerzaivskyi and Storozenetskyi, which are dominated by a rural population. Based on the last country census in 2001, there are 114,555 Romanians living in Chernivci Region (an increase from the 84,800 people in 1970). There are 76 Romanian schools (financed by the Ukrainian state) and 13 mixed schools that teach in both Ukrainian and Romanian. There are also 12 local newspapers in the Romanian language as well as three bilingual newspapers (Romanian and Ukrainian). In addition, there are 10 non-governmental Romanian national cultural organizations in Chernivci Region. 

The visited villages included Krasnoijsk, Chudej, Jizivci, Stara Krasnoshora, Nova Krasnoshora, and Prosika, populated mostly by Romanians, in Ukraine (Figure 1) and Straja in Romania.

Romanians reside in the lowlands of Southern Bukovina, which nearly corresponds to present-day Suceava County with around 635,000 inhabitants (Table 1).

In the municipality of Straja, where we conducted the study, most of the approximately 5000 inhabitants are particularly devoted to mountain agriculture, organized in small-scale farming activities including both animal breeding and crop cultivation.

After joining the European Union, the government of Romania has welcomed the issuing of a Romanian passport if someone meets the required criteria (proof that you have a Romanian relative, know some of the Romanian language, etc.). Respondents from the Ukrainian side explained that with the help of that passport they can freely travel to other European countries for seasonal or permanent employment, where salaries are much higher.

### 2.3. Data Collection

Fieldwork was carried out in the summers of 2018 and 2019. This study is part of the broader research project “Ethnobotany of Divided Generations” which, using the same method for data collection, aims to compare ethnobotanical knowledge of groups divided by the creation of a border during or after the Soviet time. The sample comprised 30 people from each side of the border, chosen pseudo-randomly, sometimes using snowball and convenient sampling methods. People were approached in streets, gardens, and fields, as well as in public places like libraries, bus stops, and markets. Oral informed consent was obtained prior to each interview. The respondents were given full freedom to talk about the subject. Only people permanently living in the study area were interviewed. Sixty interviews were conducted mainly in Romanian, but also in Russian or Ukrainian, depending on the preference of the interviewee. Participatory observation was performed during the summer of 2018. Most of the interviews in both Southern and Northern Bukovina were conducted with the help of a field assistant and facilitator, native in Romanian (for Southern Bukovina) and in the local Romanian dialect (for Northern Bukovina), who asked the questions, listened to responses, and posed follow-up questions, as Romanian is not the native language of the authors. The face-to-face interviews lasted from 20 minutes to 2 h. This study is part of a larger research project, in which questions on the use of wild plants were grouped into food, medicine, ritual and other uses modules. The food module consisted of questions specifying the use of wild plants for tea, soups, salads, main dishes and desserts, as well as any information the interviewee wanted to share about wild taxa use for culinary purposes. Oral consent was obtained prior to each interview, in which the aim of the project and the anonymous status of the interviewee were explained, as well as the condition that they could stop and withdraw all data and information at any stage of the interview. We strictly followed the ethical guidelines prescribed by the International Society of Ethnobiology [52], and the study protocol was approved by the Ethical Committee of Ca’ Foscari University of Venice.

During the interviews herbarium voucher specimens were collected. The Ukrainian voucher specimens are stored in the Nature Reserve “Roztochya” UAV with codes NB001-NB289 and the Romanian voucher specimens at Ca’ Foscari University of Venice UVV bearing codes SB001-SB094. The mentioned taxa were collected and then identified according to the local flora [53]. Taxonomic identification, botanical nomenclature, and family assignments followed the Flora Europaea [54], The Plant List database [55], and Angiosperm Phylogeny Group IV [56].

Participatory observation at local markets, shops, restaurants and near houses was performed, focusing on the wild food plants and products made from various wild parts and fruits that are sold. Participatory observations at local markets (on specific days of the week, so-called “market days”) and selling locations (e.g., on the side of the road) were carried out in order to identify supply and demand of wild products in both study areas. While conducting interviews, we also made observations of home gardens and the vegetables planted by households.

### 2.4. Data Analysis

The gathered data were organized as Detailed Use Reports (DUR), where each interviewee mentioned the use of a plant and mode of preparation (e.g., dried blueberries leaves used for tea or the leaves of horseradish as a seasoning for fermented foods) documented into the Excel spreadsheet. The spreadsheet includes informant code, language of the interview, Latin name, taxonomic family, local name (Ukrainian names were transliterated using the system adopted by the [57]; Russian names were transliterated following the general transcription rules, with the use of the webpage www.calc.ru, time of use (always, in the past, recently abandoned, recently adopted), food use, mode of preparation, source of knowledge, and comments. For all calculations only wild and semi-wild taxa were used, while unusual uses of cultivated taxa are reported only in Table 3 and the discussion.

Following the methodology of González-Tejero et al. [58], we calculated the Jaccard similarity index as:

JI = C × 100/(A + B − C)
(1)

where A is the number of recorded species in study area 1, B is the number of species recorded in study area 2, and C is the number of species common to both study areas 1 and 2.

An index value close to 100 indicates that the plant uses of the two groups are very similar, while a value close to 0 indicates that the groups are very different.

The respondents were selected using convenient sampling, in which they were approached in gardens and yards, during hay making, or at local markets. Only two people refused to speak with us, and so response rate was high; everyone else was friendly and willing to discuss wild food issues and their life experiences.

In Northern and Southern Bukovina 60 interviews were conducted (see Table 2).

In the Ukrainian study areas, Romanian is the official language in schools, while students study the Ukrainian language some hours every week. In the regional (Oblast) government offices, Ukrainian is used as the primary language, while in local village councils Romanian is spoken. In the street, Romanian and local Romanian dialects are used. As there are Ukrainians living in those villages or married to Romanians, Ukrainian with a mixture of Romanized words is used.

Furthermore, we classified the used plants into two categories based on their level of wildness: wild plants—taxa that grow spontaneously in the surrounding environment without human intervention (e.g., *Vaccinium myrtillus*); semi-cultivated plants—taxa that grow without direct human intervention, but were once planted in gardens (e.g., *Armoracia rusticana*).

Additionally, we compared our results with the wild food plant uses collected by the authors using the same interview structure among Hutsuls in the mountainous area of Bukovina in Ukraine and Romania [6].

To process the data statistically, on the request of the academic editor, we first ran a boxplot to graphically check the data. We observed that use categories of Romanians living in Ukraine are more scattered than those of Romanians living in Romania. Therefore, in order to test the observed difference between the two groups we performed a Wilcoxon test on use categories reported by Romanians living in Ukraine and in Romania. We set α = 0.05. In addition, we verified significance with a Kruskal–Wallis test using the same significance level. Both analyses were conducted using R software R (version 3.6.1).

### 2.5. Factor of Informant Consensus

The Factor of informant consensus (FIC) was calculated as the number of use citations in each category (Nur) minus the number of species used (Nt), divided by the number of use citations in each category minus one [59].

FIC = (Nur − Nt)/(Nur − 1)
(2)

The high values (near 1) indicate that the majority of respondents propose a low number of taxa for cooking in this food category, while low values (near 0) indicate the respondents’ disagreement about taxa used for certain food categories.

## 3. Results and Discussion

### 3.1. Caveats of the Study

Before discussing the results of the study we want to mention some caveats which may affect our interpretation and were considered in the following discussion. As the research is qualitative, the statistical analysis showed the following results: we performed a Wilcoxon test and a Kruskal–Wallis test, and both analyses revealed that there is no significant difference between the use categories of the two groups of Romanians living across the border. Indeed, the *p*-value for the Wilcoxon test was 0.16 and 0.092 for the Kruskal–Wallis test. Assuming that the null hypothesis for both tests was the equivalence of distributions of two groups, and given that the *p*-values are higher than 0.05, we accept the null hypothesis. We conclude that the two groups of Romanians do not present a statistically significant difference in the use of plant categories. However, we consider that the present differences, even though statistically non-significant, are important from a cultural perspective and we further focus on qualitative methods that are better suited for the analysis of cultural phenomena.

### 3.2. Contemporary Use of Wild Food Plants

In the wild food domain, Romanians living in Bukovina used 46 plant taxa (plus five cultivated taxa with uncommon uses) belonging to 20 families (see Table 3). The most used plant taxa, incorporating both sides of the border, were *Rubus idaeus* (73.3% of interviewees), *Armoracia rusticana* (50%), *Urtica dioica* (46.7%), *Rubus* spp. including *Rubus fruticosus* (45%), *Fagus sylvatica* (40%), and *Fragaria vesca* (35%).

Romanian interviewees in Romania reported 27 taxa belonging to 15 families, plus five cultivated taxa with uncommon uses, used for food. In Ukraine, Romanians named 40 taxa belonging to 18 families plus four cultivated taxa. Cultivated taxa, such as the wood of *Prunus avium*, *P. cerasus*, *P. domestica*, and *Malus domestica*, were used for smoking meat and *Vitis vinifera* leaves were used for making sarmale, which are uncommon uses, and thus we included them in the study.

In both areas of Bukovina, the Rosaceae family was the most used with 95 DUR and 101 DUR, respectively. Romanians in Ukraine used more families, including the following that were distinctive: Betulaceae (7 DUR), Boraginaceae (2 DUR), Hypericaceae (9 DUR), Plantaginaceae (1 DUR). Only Romanians in Romania used Vitaceae (2 DUR), Papaveraceae (5 DUR), and Cannabaceae (4 DUR).

**Figure 4:**
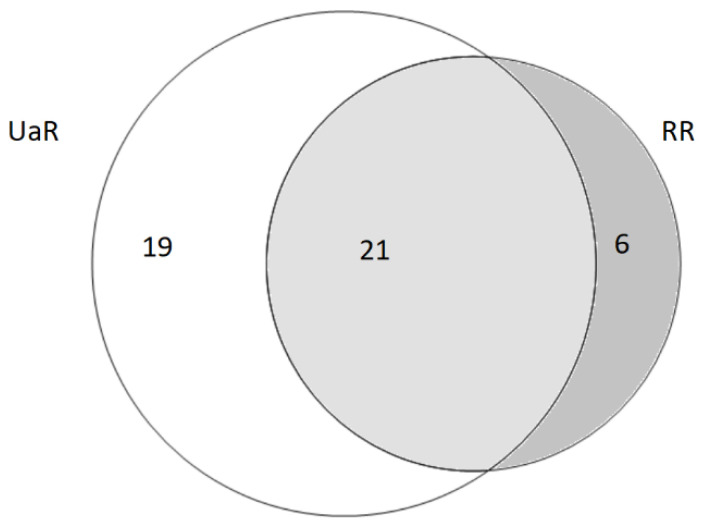
Venn diagram of overlap of wild taxa used in both study areas (UaR—Romanians in Northern Bukovina, RR—Romanians in Southern Bukovina).

**Figure 5:**
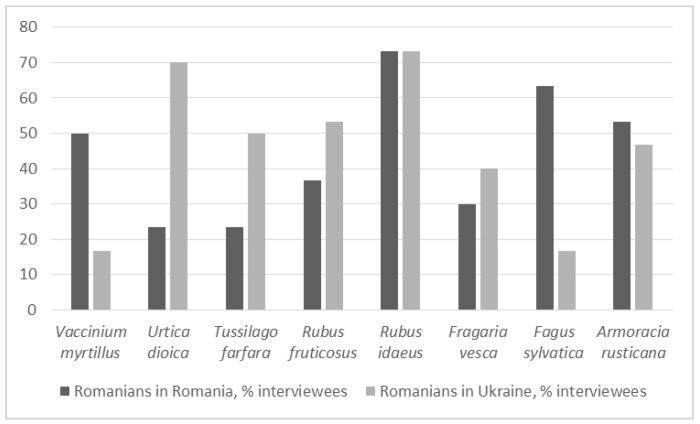
Taxa reported by the majority of interviewees.

Twenty-one taxa were common to both groups (see Figure 4), including *Rubus* spp., *Rubus idaeus*, *Rosa rugosa*, *Rosa centifolia*, *Quercus* spp., *Carum carvi*, *Fagus sylvatica*, *Fragaria vesca*, *Tussilago farfara*, *Urtica dioica*, *Vaccinium vitis-idaea*, *Vaccinium myrtillus*, *Thymus vulgaris*, and *Sambucus nigrum* (see Figure 5).

In Northern Bukovina, interviewees reported 19 taxa not named by Romanians in Romania (see Table 3), with more than twice the number of uses. These taxa included *Achillea millefolium*, *Crataegus* spp., *Hypericum* spp., *Matricaria chamomilla*, *Betula* spp. (*Betula pendula* and *Betula pubescens* which locals use as “birch sap”), and *Rumex acetosa*.

Five taxa were used only in Southern Bukovina: *Atriplex hortensis*, *Humulus lupulus*, *Robinia pseudoacacia*, *Papaver rhoeas*, and *Vitis vinifera*.

More than 70% of interviewees in both areas used *Rubus idaeus* (Figure 5), while *Fagus sylvatica* and *Urtica dioica* were used on both sides of the border but with different intensity. *Fagus sylvatica* was used four times more often in Southern Bukovina, whereas *Urtica dioica* was used three times more often in Northern Bukovina.

The division of used plant parts in Southern Bukovina was the following: fruits (119 DUR), aerial parts (63 DUR) including leaves (29 DUR), flowers (22 DUR) and petals (13 DUR), roots (22 DUR), and wood (18 DUR). In Northern Bukovina the division of used plant parts was as follows: aerial parts (125 DUR) including leaves (47 DUR), fruits (110 DUR), flowers (16 DUR), roots (21 DUR), and wood (5 DUR). Comparison shows that the most commonly used plant parts were fruits by Romanians in Romania and aerial parts by Romanians in Ukraine.

Seventeen taxa in Southern Bukovina and 26 taxa in Northern Bukovina were used by at least 10% of interviewees, with 13 shared taxa.

**Figure 6:**
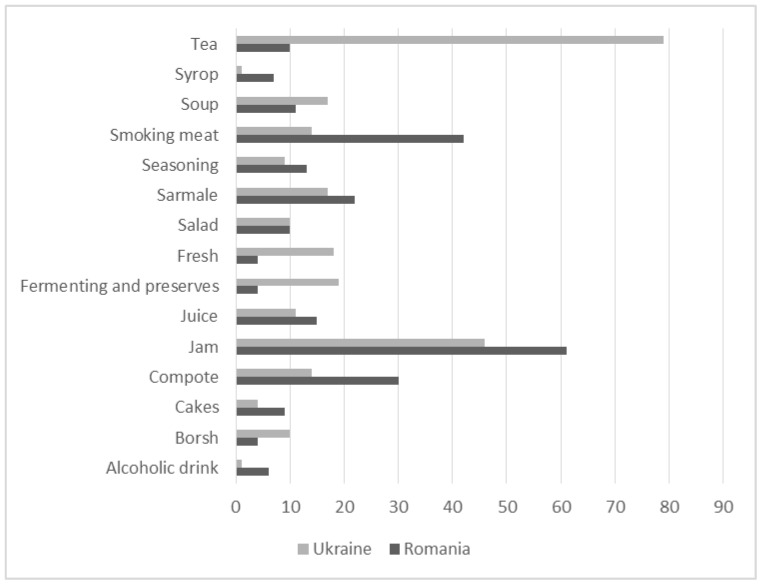
The most used food categories (with at least four Detailed Use Reports (DUR)).

Figure 6 shows the most important differences between the two samples: teas, fermented preparations, and fresh snack consumption are quite prevalent among Romanians in Ukraine, while Romanians in Romania favor wild food plant-based jams, compotes, syrups, and sarmale, as well as smoking meat.

This picture largely corresponds to the national cuisines of the respective countries [60,61,62,63], which seem to have greatly influenced the two divergent trajectories of Romanians during the past century. The predominance of lacto-fermentations among Ukrainians is possibly linked to old Slavic customs, while the prevalence teas may have been introduced via the promotion of popular teas spread through popular literature in the former Soviet Union during the Communist era [64].

In Romania, sarmale, jams, compotes, and syrups represent quintessential elements of Romanian cuisine, which historically was greatly influenced by the Ottomans. The prevalent custom of smoking meat at home in Romanian Bukovina may be linked to the persistence of this custom, which has not been weakened by food industrialization, as may have happened in Ukraine during the administration of the USSR food system. The “deficit” (a word that corresponds to the lack of high-demand products in markets, e.g., meat or sugar, during Soviet times) of meat may have contributed to a decline in the custom of smoking it among Romanians in Ukrainian Bukovina.

In Northern Bukovina the most prevalent wild food preparation was tea, while in Southern Bukovina it was jam (see Figure 6). Winter preserves, both fermented and marinated, were also highlighted. Spring soups made with four wild taxa (including *Urtica dioica* and *Rumex* spp.) were mentioned as well. Birch sap as a beverage was only named by Romanians in Ukraine because it was considered medicinal by Romanians in Romania. Salads made with wild plants were named by ten interviewees on each side of the border.

In Southern Bukovina, the fruits of *Rubus idaeus*, *Rubus* spp., *Vaccinium myrtillus*, and *Fragaria vesca*, were used for jam, as were the flowers of *Rosa rugosa*, *Rosa centifolia*, and *Taraxacum campylodes*.

The leaves of *Tussilago farfara*, *Atriplex hortensis*, *Rumex* spp., *Brassica oleracea*, *Vitis vinifera*, and *Armoracia rusticana* are used for making sarmale (from the Romanian “sarmale”; this dish consists of ground meat with rice or corn rolled in different types of the leaves, e.g., cabbage or beetroot). Sarmale was a popular preparation use of wild taxa for food. Romanian interviewees in Ukraine called this dish “găluște”, but they used fewer taxa, only the leaves of *Tussilago farfara* and *Rumex* spp.

For soups, Romanians in Romania used the young leaves of *Urtica dioica* and *Atriplex hortensis* in springtime. Romanians in Ukraine used more species for making soups, including *Urtica dioica*, *Rumex acetosa*, *Rumex* spp., and *Thymus* spp. as seasoning. *Urtica dioica* was named in both areas as an early spring food, used for making borshch (typically the term “borshch” is used to refer to a soup with meat, cabbage, carrots, potatoes, tomatoes, and beetroot with sour cream, which should be red in color). Another variety called “green borshch” is made with carrots, potatoes, boiled eggs, sour cream, and different green plants like *Urtica dioica* or *Rumex acetosa*, (thus the color of the soup is green). In Ukrainian Bukovina, *Urtica dioica* was used for stew with sour cream, in omelets and scrambled eggs, and for frying with eggs: “Nettle, in the spring, when it is young and small, we boil it and stir fry it and eat it” explained a Romanian woman born in 1939. Interviewees highlighted that they use nettle leaves only in early spring when they are tender. Fried young sprouts with sour cream were also mentioned as a “vitamin food” during spring time.

**Figure 7:**
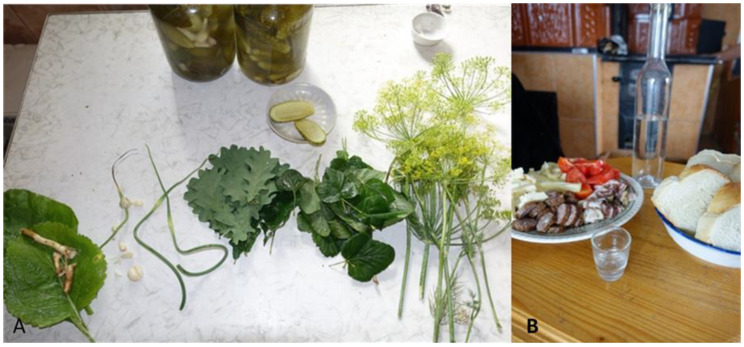
(**A**) Use of a variety of wild taxa leaves and roots (*Quercus* spp., *Armoracia rusticana*) and cultivated taxa (*Allium sativum*, *Anethum graveolens*, *Ribes nigrum*) for fermenting cucumbers; (**B**) home-smoked sausage and ham, which was smoked using the wood of *Fagus sylvatica*, *Prunus domestica*, and *Malus domestica*, sampled during a field interview. Photos by N. Stryamets: (**A**) Northern Bukovina, July 2018; (**B**) Southern Bukovina, July 2019.

In both areas, *Armoracia rusticana* was the most popular fresh root that was used for salads with beetroot and as a seasoning for pickles and fermenting winter preserves (Figure 7). Romanians in Northern Bukovina used the young leaves of *Urtica dioica*, *Plantago major*, and *Taraxacum campylodes* for salads, whereas Southern Bukovinians only used *Armoracia rusticana*. It was referred to as semi-cultivated because it grows everywhere, including home gardens.

The dominant use of wild food for salads was that of grated roots of *Armoracia rusticana*, which was named by 47% of Romanians in Romania.

### 3.3. Wild and Cultivated Plants for Smoking

*Fagus sylvatica* was used in both areas for smoking meat. The wood of *Prunus avium* (9 DUR), *Malus domestica* (2 DUR), *Prunus cerasus* (4 DUR), and *Prunus domestica* (8 DUR) was also used for smoking meat by Romanians in Romania. Meat smoking was explained as a way “for meat to last long”, because when you raise your own pigs, you have to be able to store the meat. In earlier times, when freezers were not available, smoking was an important preservation practice for local livelihoods.

In Northern Bukovina, besides *Fagus sylvatica*, the wood of *Carpinus betulus* was used for smoking meat. In addition, in both areas of Bukovina, fruit trees branches were mentioned as “adding taste and color”, which was explained as: when you prune trees in your garden in the autumn and spring, you keep the branches for smoking purposes. As in Southern Bukovina, the wood of *Prunus avium* (5 DUR), *Malus domestica* (1 DUR), *Prunus cerasus* (4 DUR), and *Prunus domestica* (4 DUR) was also used for smoking meat in Northern Bukovina. The wood of cherry trees gives a reddish color to the meat, and the more cherry wood you burn, the deeper the color of red you achieve, explained a retired interviewee. Smoking was mentioned as an important practice for the long preservation of pork meat. In Southern Bukovina, only one interviewee mentioned the practice of cheese smoking. Smoking practices and the use of both wild and cultivated taxa were not mentioned by researchers either in Romania [4,33] or Ukraine [7,24]. Historical sources show that this practice of preserving meat was popular among inhabitants of the Carpathian Mountains [34,43].

### 3.4. Plant-Based Beverages

Alcoholic beverages were only mentioned in Southern Bukovina. This included rachiu, which is prepared with *Carum carvi* and home-distilled alcohol (see Figure 7). Aromatized alcohol drinks were also popular. The fruits of *Rubus idaeus* and *Vaccinium vitis-idaea* macerated in alcohol were mentioned by Romanians in Romania. In addition, the fruits of *Vaccinium myrtillus* were used for an alcoholic drink called “afinătă” (from the Romanian afină—blueberry), which added colored and aroma to the alcohol. Four interviewees also mentioned homemade beer, which was made from the flowers of *Humulus lupulus* or sometimes the flowers of *Sambucus nigra*.

Syrups made from *Rubus idaeus*, *Fragaria vesca*, *Rosa rugosa* or *Rosa centifolia*, and *Rubus fructicosus* were mentioned by Romanians in Southern Bukovina. Their use was intended for weekly fasting days: “Usually when you fast on Wednesdays and Fridays you eat raspberry syrup with bread”, explained a man born in 1965. In both study areas, Romanians fasted on Wednesdays and Fridays and during four fasting periods throughout the year. Meat and dairy products are not allowed to be eaten on those days, and thus syrups and jams made from wild fruits are consumed by locals. The use of food plants for fasting, rituals and ceremonies needs to be more thoroughly studied.

In Southern Bukovina, the fruits of *Rubus idaeus*, *Rubus* spp., and *Vaccinium myrtillus*, and the flowers of *Sambucus nigra* were used for juice preparations. Interviewees also explained that the flowers of *Sambucus nigra* are used for making a fermented drink called “socată” (from the Romanian word ‘soc’—*Sambucus nigra*). The flowers of *Sambucus nigra* are collected, and then 10 flower inflorescences, 1 kg of sugar and a lemon are added to 10 l of warm water and a teaspoon of dry yeast; after one day this mixture is bottled and stored in a cool place. The dried flowers also were named as suitable for preparing “homemade beer”. The beer is lacto-fermented and may contain a small amount of alcohol. The same use was described in Roztochya (Western Ukraine), but it is called “sparkling drink” [7].

### 3.5. Homogeneity and Inhomogeneity of Wild Plants Use for Food Preparations

As the analysis of Factor of Informant Consensus reveals (Table 4), in Southern Bukovina among Romanians consistently used the same taxa for smoking of meat, seasoning and sarmale, while for tea and borshch more diversity was used. In Northern Bukovina, Romanians homogeneously use plants for jam and fermentation, while there was disagreement, meaning they made a not homogenous use of plant taxa, for alcoholic drinks, syrup and fresh food.

### 3.6. Past Uses of Wild Food Plants

In Romania, interviewees explained that in the past more forest products were used, mainly to generate income. In Northern Bukovina, their function as a food substitute for “deficit” products was mentioned, e.g., coffee was difficult to buy and so locals made coffee at home from the roots of *Arctium lappa* and *Taraxacum campylodes*. The decline in the use of wild plants for food was mentioned in both areas of Bukovina. Two interviewees in Northern Bukovina mentioned that in the past their grandparents used the leaves of *Urtica dioica* for making tea and soup. Likewise, tea made from *Achillea millefolium* and *Matricaria chamomilla* were named by 10% and 6% of interviewees, respectively, as past uses, again by their grandparents.

Compote and jam prepared from *Vaccinium myrtillus* and *Rubus idaeus* were mentioned as recently abandoned food practices by one interviewee in Southern Bukovina. Six percent of interviewees named jam made from *Rosa rugosa* as a recently abandoned practice. Smoking meat with the wood of *Fagus sylvatica* was also mentioned by one person as a recently abandoned practice. Romanians in Romania did not mention past uses of wild food plants. Participatory observation in both study areas indicated that restaurants currently offer dishes with wild mushrooms and forest fruits, and they even sell dried wild forest fruits. In Southern Bukovina, local restaurants serve wild mushroom sauce, and desserts with wild forest fruits (blueberries and raspberries) were offered on the menu. In Northern Bukovina, a variety of forest fruits and plants were sold at local markets. In small local restaurants, tea made from forest fruits and plants was served, while blueberries were added to desserts, which in the description indicated that they were “forest” blueberries.

The use of wild food in our study populations plays a food supply role, as well as a culturally important role. For example, in Southern Bukovina wild foods were used to help generate income in the past and they are currently important in everyday cuisine.

### 3.7. Wild Edible Forest Plants

In both groups of Romanians, wild forest taxa were the most used, including *Rubus idaeus* (Romanians in Northern Bukovina (UaR) 40 and Romanians in Southern Bukovina (RR) 43 DUR), *Rubus* spp. (UaR 19 and RR 21 DUR) including *Rubus fructicosus* and *Rubus caesius*, *Vaccinium myrtillus* (UaR 7 and RR 23 DUR), *Fragaria vesca* (UaR 15 and RR 15 DUR), *Vaccinium vitis-idaea* (UaR 2 and RR 3 DUR), *Fagus sylvatica* (UaR 5 and RR 19 DUR), and *Urtica dioica* (UaR 26 and RR 7 DUR) (see Figure 6 and Table 3). These results are unusual as the local landscape of the region consists predominantly of flat lowlands with agricultural fields (Figure 2), and thus the distance to the forest and the fact that “most of the forests are situated in the mountains” were not seen as an obstacle. “We buy blueberries, raspberries and a variety of mushrooms at the market because those products grow in the forest up in the mountains”, highlighted a Romanian woman from Northern Bukovina born in 1966. Our findings, therefore, show that forest products and forest taxa still play an important role in local ecological knowledge (LEK), which is consistent with Pieroni and Sõukand [24].

“We use forest berries [fruits]: blueberries, raspberries, blackberries, but they grow in the Carpathians, and you have to go far to harvest them”, explained a young Romanian woman from Northern Bukovina born in 1983.

The better economic situation in the region is now allowing locals to buy forest products instead of collecting them themselves: “Before we were going to harvest blueberries, we started to go to the forest at 3 am; it was so hard and so time-consuming to collect blueberries. Now we buy them at the market”, explained a Romanian woman born in 1947 in Northern Bukovina. “This year a friend brought me birch sap because it is too far to go to get it in the forest”, stated a retired woman from Northern Bukovina. “We buy forest mushrooms and berries, they are tasty”, asserted a retired Romanian woman in Ukraine. Romanians in Romania explained that in the past they used the forest fruits *Vaccinium myrtillus*, *Fragaria vesca*, and *Vaccinium vitis-idaea* to generate additional income in times of scarcity and economic crisis. However, nowadays they buy forest berries at the market or in shops.

Romanians in Ukraine also mentioned purchasing wild forest fruits, but mostly because “they grow far away in the mountains and one has to have time to harvest them”, so it is much more convenient to buy them at the market.

Participatory observation at local markets revealed the sale of home-made dairy products and forest products (wild blueberries, mushrooms) in Northern Bukovina, while in Southern Bukovina it was mostly produced goods. The reason for this difference could be that in Romanian Bukovina milk collection from homes is still present (which was observed during fieldwork), so there is no need to process and sell milk yourself. Legislation in Ukraine allows the selling of forest products by locals, but there is currently discussion on banning the sale of dairy products by locals [65]. By law in Ukraine, the harvesting of forest products with commercial purposes has to be carried out with a special permit, which is a tax-based payment to local forest enterprises; however, this is not widely practiced due to bureaucratic complexity [14]. More active enforcement of this legislation may influence the use of wild forest products due to a reduction in the number of collectors and an increase in prices (to offset the additional cost of the permit). In Romania, the collection of forest products is free of charge for personal consumption, while it requires authorization for commercial purposes (according to article 58 of the Forest Code).

In both study areas wild plants including forest products continue to be used in daily cuisine, while in Southern Bukovina interviewees explained that those products also played a role in income generation in the past. Therefore, nowadays, in both areas the purchase of wild forest products was mentioned much more often than collecting them in the wild. This economic influence on wild product use is consistent with the findings of Stryamets [22], in Smăland, Sweden, where locals buy rather than collect non-wood forest products (NWFPs). So here, economic factors have highly influenced the use of forest products: first locals actively used them and sold them for additional income, but as the economic situation has improved, people have stopped selling them as they have money to buy them at local markets. This is the first step toward losing this LEK as children do not learn how and where to collect them; the next step involves people stopping cooking them (if these products are not available in shops). The situation in Swedish Smăland demonstrates that LEK has been eroded because of the healthy economic situation [7,22] and the consequent loss of the practice of foraging for NWFPs. One interviewee from Southern Bukovina explained that border creation reduced his opportunities to go to the forest, because most of the historical forest of Straja now belongs to Northern Bukovina; however, forest cover in Southern Bukovina is 63% compared to 37% in Northern Bukovina.

### 3.8. A Childhood Delicacy: The Past to Present Fashion of Dainty Foods

Rose syrup and jam made from the flower petals, minus the white part, of *Rosa rugosa* and *Rosa centifolia* were considered delicacies and used on special occasions. “In the past we used a specific rose for sweets and it was a delicacy; only respectable people made sweets with green walnuts and sweets with roses; they were for guests [Means that sweets with roses were used for only special occasions for guests]. I know that my mother made syrup and jams and locked them up [to be used only on special occasions and kept away from children]; they also made sherbet. The little ones went to the forest for raspberries and sold them in the town center to have money to buy school supplies, uniforms”, explained a Romanian woman born in 1957. In the Ukrainian part of Bukovina, rose jam was used for religious celebrations as an additive to cakes and doughnuts. “We also prepare raw jam with rose flowers; what tasty buns you get with it! They are delicious”, highlighted a Romanian woman from Ukraine, born in 1953. Nowadays, for confectionery purposes, one can buy rose syrup in specialty shops, for UAH 2700 (80 Euro) per kg, and the syrup is used in fashionable restaurants and by elite cakes producers. Historical Ottoman influence introduced a few elements which can still be found among Romanians living in Bukovina. One recipe is rose jam made with the petals of *Rosa rugosa* and *Rosa centifolia*. This recipe can also be found among other groups that were under Ottoman control, such as Hungarians [66] and Bosniacs [67]. Interestingly, this product was almost exclusively mentioned by Romanians in Romania. In addition, tea is also sometimes made from the petals of these roses. Both jam and tea can also be found in shops in Romania.

Another Ottoman element is sarma [68], which is locally called sarmale (mainly in Southern Bukovina) and găluşte (mainly in Northern Bukovina). This dish consists of meat and rice wrapped in leaves. For this purpose, the leaves of *Tussilago farfara*, *Atriplex hortensis*, *Rumex* spp. *Brassica oleracea*, *Vitis vinifera*, and *Armoracia rusticana* were used. One interviewee used horseradish leaves: “I make sarmale with horseradish [*Armoracia rusticana*], and loboda [*Atriplex hortensis*]. [Horseradish leaf] is placed between sweet-leafed cabbage rolls. We also do it with grape leaves”, highlighted a Romanian woman born in 1955. All the mentioned taxa are common plants for this preparation; however, they were not previously reported in Romania [68].

The fact that homemade alcoholic drinks were mentioned in Southern Bukovina but not in Northern Bukovina may be the result of Soviet propaganda and punishment for preparing moonshine and homemade alcohol. However, alcoholic tinctures with a variety of forest fruits were used for medicinal purposes by Hutsuls in Bukovina [6].

### 3.9. Comparison with Hutsuls

**Figure 8:**
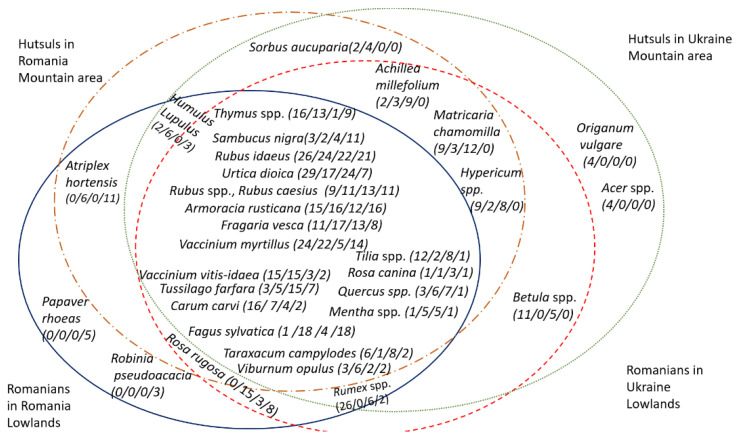
Wild food taxa used by the four groups (mentioned by at least three interviewees). The numbers in brackets correspond to interviewees that named the plant taxa (A/B/C/D), where A (dotted green circle)—Ukrainian Hutsuls, B (dotted and dashed brown circle)—Hutsuls in Romania, C (dashed red cycle)—Romanians in Northern Bukovina Ukraine, D (solid blue circle)—Romanians in Southern Bukovina. In the central part of the figure the taxa used by all four groups are represented.

Comparison with Hutsuls living in Bukovina reveals that 19 taxa are used by all four groups (see Figure 8). Hutsuls in Ukraine and Romania use the same taxa as Romanians in Ukraine for recreational tea: *Arnica montana*, *Matricaria chamomilla*, and *Achillea millefolium*. Hutsuls in Romania and Ukraine and Romanians in Romania, however, use *Viburnum opulus* and *Humulus lupulus* for this purpose (see Figure 8).

The use of *Plantago major* as a tea has also been documented among Hutsuls in Ukraine, but not among Boykos [7]. Cross-border comparison of wild plant uses shows that *Equisetum* spp., *Acer* spp., *Aronia melanocarpa* (Michx.) Elliott, *Picea abies* (L.) H. Karst., *Sorbus aucuparia* L., and *Chenopodium album* L. were used only by Hutsuls.

Cross-cultural analysis in Ukraine reveals 29 shared wild taxa, whilst five of them (*Arctium lappa*, *Plantago major*, *Crataegus monogyna*, *Trifolium* spp., and *Betula* spp.) are specific for Northern, but not Southern Bukovina. In Romania, cross-cultural analysis of food plants used by Romanians and Hutsuls in Southern Bukovina reveals the use of 22 shared taxa.

The Jaccard similarity index (JI) calculated for Romanians on the two sides of the border is 45.65. The JI for taxa named by at least 10% of interviewees is 43.33. Comparison with other studies [4,6] shows that the calculated similarity index with regard to wild taxa used for food within the Hutsul ethnic group across the border is 55 when considering all taxa and 44 when considering only those taxa mentioned by at least 10% of interviewees (Table 4).

Hutsuls and Romanians living in Romania share 22 taxa (including cultivated ones) used for food, with a JI of 53.66.

**Figure 9:**
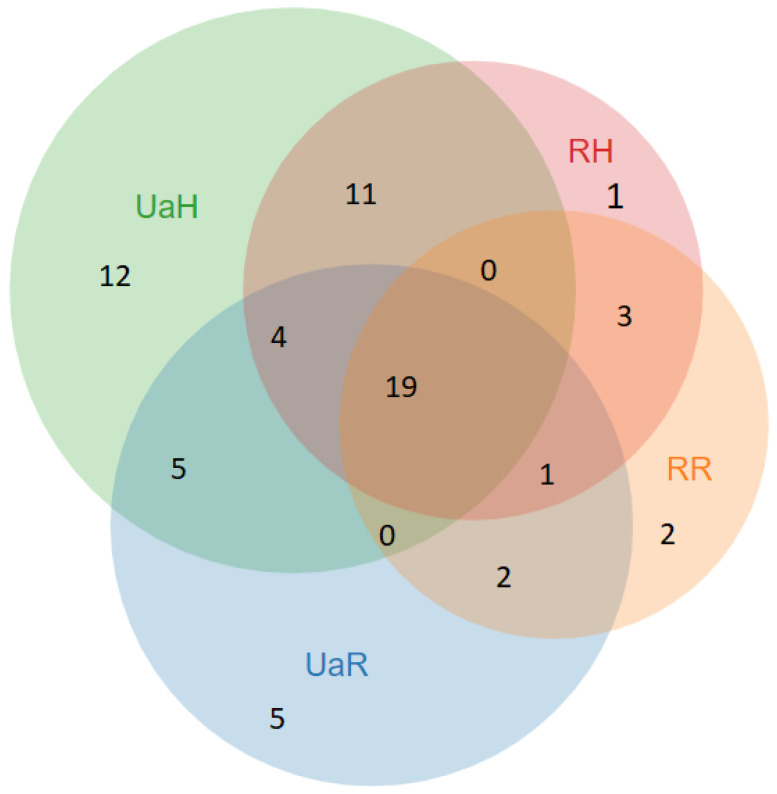
Venn diagram of the comparison of food taxa uses by the four groups. (RH—Hutsuls in Romanian Bukovina, RR—Romanians in Romanian Bukovina, UaH—Hutsuls in Ukrainian Bukovina, UaR—Romanians in Ukrainian Bukovina).

In Ukrainian Bukovina, both Hutsuls and Romanians use a greater diversity of taxa, whereas Romanian Bukovina Hutsuls and Romanians use fewer taxa but with greater intensity (based on DUR) (Figure 9).

The calculation of JI without cultivated taxa revealed even more similarity (Table 5) in uses within a country: JI for Romanians and Hutsuls in Romania is 61.11, and for Romanians and Hutsuls living in Northern Bukovina it is 52 (data on wild plant taxa used for food by Hutsuls is from [6]).

The homogenization of LEK among Romanians living in Ukraine and Hutsuls, which live in different environments but within same country, may have been due to Soviet influences as discussed by Pieroni and Sõukand [5], e.g., propaganda of wild plant use at schools as the availability of local foods was inadequate. An older Romanian interviewee, who was born in Ukraine in 1953 and worked at the library, explained that she and a school biology teacher have made an excursion path into the forest for two km where they explain to children the medicinal and food plants that growth there. “It feels so good when kids go there and ask about the plants and collect plants. It is so important to teach kids”, explained the retired librarian.

**Figure 10:**
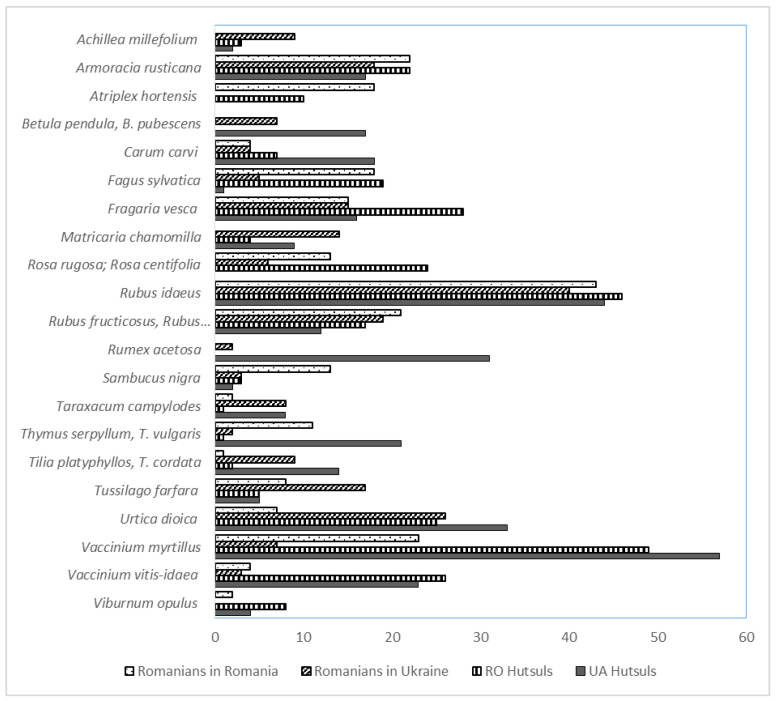
Number of uses of the most used taxa by the four groups (more than 10 DUR).

Figure 10 shows, in particular, the prevalence of *Achillea*, *Betula*, *Matricaria*, *Taraxacum*, *Tilia*, *Tussilago*, and *Viburnum* spp. food uses among Romanians in Ukraine and of *Atriplex*, *Rosa*, *Sambucus*, and *Thymus* spp., as well as of *Vaccinium myrtillus*, among Romanians in Romania.

These remarkable differences correspond again, following the pattern we outlined for plant preparations, to the most prototypical wild food plants occurring in the foodscapes of Ukraine and Romania: *Betula*, *Taraxacum*, *Tilia*, and *Viburnum* are particularly crucial plants in the Eastern Slavic and wider post-Soviet wild food domain [33,69,70,71], while *Atriplex*, *Thymus*, and *Vaccinium* spp. are salient ingredients of the Romanian foodscape [32,37]. These data show again how the wild foodscapes of Romanians have been diversely influenced in the past century by national cuisines on both sides of the border, possibly via intense contact and cohabitation with neighbors (Ukrainians in Ukraine and Romanians in Romania, respectively), facilitated by intermarriages and the celebration of an atheistic, non-religious ethos which Communism provided.

### 3.10. Attitudes and Perceptions

Cross-border Bukovina is very diverse and multifarious. Nice lawns with magnolias and decorative trees neighbor yards with home gardens growing a variety of crops; a huge jeep car is followed by horses. In a local pizza place a young woman buys pizza for the family dinner (instead of cooking it herself which is the more common and much cheaper practice in rural areas, e.g., for the price of three pizzas one can feed the family for a week), while in the next house we see home-baked bread and a cow kept for milk for the children. The interviewees explained that most of the young people are abroad for seasonal work. Women or grandparents stay at home to take care of the kids. Visually, the differences in the level of income are seen very clearly, with expansive home and fence decoration and a wooden roof house close by.

### 3.11. “There Is No One to Cook for” in Southern Bukovina

The demographical situation in both Southern and Northern Bukovina regions over the past several years has changed dramatically—the younger generation has moved to the cities or richer areas of the European Union. Elderly individuals are the ones who remain in the region. There are a few current trends: (1) the younger generation sends back money to build houses; (2) the men of the household leave for seasonal jobs and then return; and (3) younger individuals move out and never come back. During our interviews we noted all of these trends. Some people, including several middle-aged men who worked in Germany, explained that they have tried “the hard living in a foreign land” and will eat “bread and water”, but they would never go back. Others explained that because there is nowhere to work in Bukovina they (or at least someone from their family) have to go for seasonal work to earn money. There were cases in which children were left with grandparents, while parents went abroad and “talked with and educated kids via the Internet”, but they physically did not see them for years. We also observed the huge newly built houses in the area that stand empty (see Figure 3 and Figure 4). All this trend has influenced the use of wild plants for food. The youngsters have left on their own, there is a missing chain (parents are abroad and it is not possible to learn the way of wild plant use via the internet) in the transmission of knowledge. Economic influence also reduces the need to collect wild plants and the last trend the absence of children close to grandparents. A Romanian woman, born in 1947, explained that because there are no younger children or grandchildren left with her, she has no one to cook wild foods for any more: “I have bought one liter of blueberries. Now I live alone, and I have no one to cook for. Before I was cooking a lot; now my kids left, so I don’t need so much for myself”.

### 3.12. “Like in Italy” in Northern Bukovina

In Northern Bukovina besides the emigration trend, we observed how the influence of experience local’s gain abroad was transmitted to local cuisine. Meanwhile, the locals sometimes perceived the wild food plants as “famine food or poor food”. A woman born in 1928 explained that there was no Holodomor (the artificial famine) like in other parts of Ukraine, so they do not use many wild plants for food. This indicates that there is a view of wild plants as famine foods, which is in line with previous studies [7,10].

“This is păpădie (*Taraxacum campylodes*), we use it for salads, like in Italy”, explained a Romanian woman born in Ukraine in 1966. “I spent 8 years working in Italy; I just came back. I buy tea, I don’t collect it”, declared a 54-year-old man from Northern Bukovina.

“I have tea from Italy, I don’t drink tea from local herbs”, stated an older Romanian woman in Northern Bukovina. “We are not poor to drink herbal tea”, explained a retired Romanian woman. Nowadays some respondents see wild food as a famine or peasant food, which is in agreement with many findings from contemporary Europe [10,28].

Two interviewees explained that they do not collect *Origanum vulgare* nor buy bay leaves (*Laurus nobilis*) because they obtain them from Italy.

Among Romanians in Ukraine, a widespread response was that they buy tea, instead of collect it from nature: “We don’t make other teas, the majority we buy. There are big and small packages, I think they are called gruzinskii”, explained a Romanian woman born in Ukraine in 1939. One pensioner, a Romanian women living in Ukraine, asserted that “we are not poor, we have tea and coffee from Italy”, so there is a view of wild food as “poor” food, in contrast to food coming from abroad (considered elitist, which has Soviet roots when products from abroad could only be found in specific shops in capital cities, and those who could afford them were seen as wealthy).

Globalization and the homogenization of cultures, languages and natural resource use is a trend nowadays [40,41].

In addition, in both of our study populations, results show that an open work market has negatively influenced wild food use, including its abandonment and decline, due to:

1. The emigration (both seasonal and permanent) of young and middle-aged individuals from border areas to other European countries. New knowledge and practices of wild plant use as a result of traveling and working abroad, e.g., “Salad like in Italy”, and “I don’t collect herbs from here [the surrounding environment], I have tea from Italy”.
2. The idea of “no one to cook for”, which involves a reduction in the use of wild foods due to depopulation of rural border areas by younger generations, leaving only pensioners in the region. This is about unwillingness to invest time in elaborate recipes (or hard-to-find ingredients) because of the lack of loved ones to motivate such an effort.
3. The decline in the use of wild plants because of replacement by mass market products, e.g., “I have *Origanum* from Italy; I don’t collect it anymore”.
4. The valorization of forest products and higher income level which allows people to buy forest products instead of harvesting them from the wild, e.g., “We buy blueberries”.

Our results demonstrate the use of wild plants was as a part of cultural identity and self-actualization. It is shown with the childhood memories, or use of wild plants for specific, time-requiring dishes (e.g., smoking meat).

**Figure 11:**
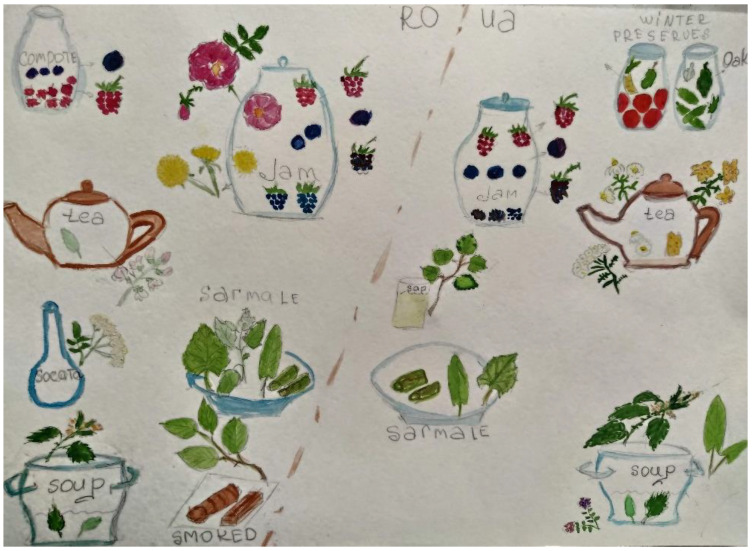
The dining table of Romanians divided by a border, showing the main differences. Drawings by S. Stryamets.

Living in same ecological conditions, but with different socio-economic scenarios, we discover differences in the use of wild foods by the same ethnic group (Figure 11). Problems with infrastructure and the lack of job opportunities in Ukrainian Bukovina compel locals to be more dependent on available wild food resources, while in Southern Bukovina the income-generating function of wild foods is confined to the past. The use of wild plants that are strongly associated with local cultural identity and are present in local culinary traditions [72], the maintenance of wild taxa used as childhood treats, and specific local culinary recipes (e.g., smoking meat, afinată, socată, sarmale and green borshch) are less likely to disappear from local cuisines. However, our results demonstrate the strong influence of an open work market and social migration on the use of wild plants. Rapid changes in lifestyle and habits are one explanation for the abandonment of wild food use, which is consistent with Serrasolses et al. [72].

The majority of food taxa used by Romanians in Ukraine, especially for making tea, can also be used for medicinal purposes. This food–medicinal continuum when comparing the two groups was also observed in Sõukand and Pieroni [6].

In Bukovina, 51 taxa (46 wild taxa plus 5 cultivated taxa with uncommon uses) were used for food, compared to 40 taxa recorded by Sõukand and Pieroni [4]. This number, however, is lower than the 70 taxa recorded in Polissya Region [24], yet higher than in Roztochya with 26 taxa [7], or the 44 taxa used in Maramureș [33].

Romanians in Ukraine used four wild taxa (*Urtica dioica*, *Rumex acetosa*, *Rumex* spp., and *Thymus* spp.) for soup preparations, while Romanians in Romania used only two taxa (*Urtica dioica* and *Atriplex hortensis*). In other parts of Ukraine the number of plants use for “green” soup reaches 21 taxa [26]. In Southern Bukovina, *Atriplex hortensis* is not seen as wild because it grows in gardens, similar to that of *Rumex acetosa* in the area of Roztochya, where this taxon grows in yards and gardens [22].

Birch sap was widely used in Northern Bukovina as a refreshing drink and also fermented for winter time. It is popular in other parts of Ukraine and Eastern Europe as well [5,71], and thus could have a Soviet origin.

The flowers of *Robinia pseudoacacia* were mentioned as a famine food during times of scarcity in Polissya Region [24]. The possible attenuation in use of *Atriplex hortensis* by Romanians in Northern Bukovina may be related to the fact that it is seen as a famine food [73]. The prevalent use of recreational tea by Romanians in Ukraine could have Soviet roots, as teatime and herbals tea were promoted during this era. Sweets being the dominant preparation mode in Southern Bukovina may be explained in a couple of ways: the “deficit” of sugar during economic crises and sweets being seen as rich people’s food, and the Turkish influence of using jams and sweets. The difference in the use of winter preserves and fermenting may be due to the unstable economic situation in Northern Bukovina, which forces people to have their own food security system for winter time and times of uncertainty.

The taste of wild foods was also one of the factors influencing the use of plants, e.g., jam made from *Rosa rugosa* is considered to be a delicacy, and nowadays there is a trend toward the valorization of products made from *Rosa rugosa* in both areas of Bukovina. Studies have demonstrated that taste is culturally developed [74]. Therefore, in our case taste was not the main factor influencing wild food use [72].

The use of wild foods not only serves nutritional and safety-net functions [75], but it is a source of self-actualization and contributes to well-being [42], e.g., “I cook as my mom and grandma used to cook, using wild herbs”. Complex human-nature relationships are formed with the use of wild foods growing in the surrounding landscape, creating a unique local cuisine. However, socio-economic and governance systems have produced differences in uses by the same ethnos in the same environment. An improved economic situation leads to a decline in the use of wild taxa for food, as well as harvesting practices (purchasing food items at the market instead of collecting them from the surrounding landscape) and cooking practices, e.g., “it is hard to cook and there is no one to cook for”.

Differences across the border are remarkable: along with the use of 21 shared taxa, we recorded 25 different wild food taxa. The most remarkable differences between the two sides were related to mode of preparation. We suggest that those differences are the result of the influence of intense contact and cohabitation with neighbors (Romanians on the Southern Bukovina side and Ukrainians on the Northern Bukovina side). In Romania, Bukovinians were part of a larger group and did not have to preserve any ethnic belonging and cultural identity. While in Northern Bukovina, Romanians had to somehow integrate with Ukrainians, yet remain Romanian in order to save their cultural identity. In Northern Bukovina, interviewees mentioned that their children were studying in Ukrainian universities; even though schools in the region are Romanian, they study Ukrainian and some teachers are Ukrainian. The peaceful coexistence of these two ethnic groups has resulted in intermarriages and close connections. Interviewees also highlighted Moldova as a state with a similar language (they use Romanian with a Cyrillic alphabet) and education in Moldavian institutes during Soviet times.

Therefore, the factors possibly influencing local cuisine and the use of wild food plants include:

1. The border that has divided Bukovina for the last 80 years has resulted in different socio-political scenarios and thus in different food behaviors and the use of wild plants in local cuisine. Long-standing borders have had a tangible effect in shaping divergent wild plant foodscapes, since the respective national states of Northern and Southern Bukovina, and possibly different neighbors, have shaped the Romanian dining table in different ways.
2. Intermarriage and neighbor influence. At least three interviewees in Northern Bukovina explained that they were married to a Ukrainian, which may have influenced their food behavior: “I cook green borshch as Ukrainians do” and “My daughter-in-law is Ukrainian, from Kolomyya; she cooks 12 dishes for Christmas Eve, and she teaches me”. Another male interviewee stated: “In the neighboring Novoselecjk region they ferment apples and melons, but we don’t”.
3. Economic differences: in order to smoke meat one has to be able to afford it, and during Soviet times meat was “deficit”, while during the economic crisis between 1990 and 2000 rural individuals could not afford it. In contrast, herbal teas are easily affordable, and among Romanians it was twice as popular in Northern Bukovina as in Southern Bukovina. The use of birch sap clearly has Soviet roots as it was promoted as a “healthy and tasty drink”. Winter preserves, as a way to survive during economic crises, are still popular in Northern Bukovina as a food security practice.
4. Market influence: Romanians live in close proximity to other ethnic groups with whom they interact at local markets; for example, participatory observation in Northern Bukovina has revealed the possible exchange of culinary experiences at “babushka” markets [65].
5. The media: local newspapers and television food shows may also influence local cuisine, as recipes for different dishes have been published in the local press.

The latest UN report [76] highlighted that in terms of global food issues locally grown foods and the use of wild foods from local landscapes are the answer to the challenges that humankind currently faces. That is why documenting and discussing LEK on wild food use is a crucial and timely endeavor.

## 4. Conclusions

Contemporary use of wild taxa by Romanians in Bukovina Region was diverse and different across the border. Romanians in Romania use fewer taxa in a less intensive way, while Romanians in Ukraine use more diverse species and have more diverse uses, including those that support food security in winter time. In the wild food domain, Romanians living in Bukovina used 46 plant taxa (plus 5 cultivated taxa with uncommon uses). Despite the flat landscape with extensive agricultural fields, Romanians on both sides of the border used forest taxa the most as wild food.

The limitation of the study is that the two groups of Romanians do not present a statistically significant difference in the use of plant categories, therefore the qualitative results of this study represent the contemporary use of wild plant taxa for food purposes, showing 566 DURs.

Limitations of the study: in both case study areas the women were the dominating (80%) interviewees; therefore we consider this division as relevant to these conditions (in the gender strictly of the older generation the women dominated) and qualitative methods. As in both areas the help of the field assistant was used, some small details of the interviews might be lost in the translation.

Our results show that the use of wild food plants in Bukovina is more similar between ethnic groups (Hutsuls) within the same country (Romania or Ukraine) than within groups across the border (Romanians living in Romania and Ukraine), which supports the main hypothesis of the DiGe (Divided Generations project) that socio-political scenarios influence the use of wild foods. Indeed, cross-border studies can greatly contribute to enhance our understanding of the impacts of political (and consequently social and economic for instance) contexts on the local ecological knowledge. This can be crucial to shape effective policies according to the perceptions and management of local resources adapted to different political contexts. Such policies can be especially important in areas rich of biological and/or cultural diversity, which therefore would require context-based conservation and valorization policies.

The 80 years spent living under different governance systems has resulted in different ways of using wild plants in daily cuisine. The use of food plants by Romanians is more markedly shaped by intermarriage and neighbor influence (through media and local markets), as well as the recent phenomenon of globalization/open work market, as a result making use of some plants similar to those found in countries where interviewees worked (e.g., Italy). The improved economic situation (the possibility of buying produced food instead of collecting it in the wild) and the availability of industrially produced goods have also influenced current uses, e.g., decrease in the use of wild plants in everyday cuisine. Depopulation of rural areas and a growing disconnect with nature has also significantly influenced the decline in the skills and practices of wild plant use. In addition, the valorization of forest products and a higher income level allows for buying forest products instead of harvesting them from the wild.

This research gives the opportunity to understand the dynamic structure of local cuisine, influences by the socio-political scenarios, globalization, intermarriage and co-existing of different cultures, and media influence, market flow of products as well as the economic situation which allows limitation of wild plants use. Based on these results, the prognoses of more disconnection with nature, less wild plants use for everyday cuisine could be reached. Therefore, the internal global crises events, e.g., the COVID-19 pandemic or issues with food supply in the remote areas might increase the wild plant use for food.

Complex human–nature relationships are formed with the use of wild foods growing in the surrounding landscape, creating a unique local cuisine. There is a need for further research into how socio-political scenarios under conditions of globalization, in terms of the flow of people, investment, market product and knowledge, influence LEK and food habit changes, focusing on biocultural values and dynamics of local cuisine.

The use of wild food as a self-actualization and contribution to well-being also requires future studies. Further research is needed to explain food uses for cultural purposes; for example, religious celebrations. Another issue that was frequently mentioned by locals and seen as an important culinary practice, and thus needs more thorough analysis and consideration, including cross-border comparison, is food habits connected to ceremonies and ritual behaviors.

In the framework of recent debates on the significance of wild food plants, especially during the Covid-19 crisis, current research is vital in terms of the application of LEK and understanding the driving forces of wild plant use. The next issue that demands research is home-grown foods as a food security measure. During the participatory observations and during interviews, locals named the food gardens close to homes as an important source of both supplementary food and “healthy self-growing food”. The use of wild foods as ethnomedicine for humans and for animals is another important subject for our future research.

## Figures and Tables

**Table 1:** The environmental and socio-economic characteristics of the study areas [48,49,50,51].

Characteristic	Northern Bukovina (Lowlands)	Southern Bukovina (Straja)
Climate type	Dfb	Dfb
Annual precipitation	601 mm	673 mm
Altitude	350 m a.s.l.	520 m a.s.l.
Average temperature	−5 °C January 19.6 °C July	−5 °C January 17.6 °C July
Population density	110 people/km^2^	111 people/km^2^
Forest cover	37%	63%

**Table 2:** Socio-economic data of the interviewees.

Characteristic	Northern Bukovina	Southern Bukovina
Number of interviews	30	30
Average age of the interviewees	63	61
Eldest interviewee	Born 1930	Born 1928
Youngest interviewee	Born 1983	Born 1987
Gender distribution	24 female (80%), 6 male (20%)	24 female (80%), 6 male (20%)
Education level	45% primary education 37% secondary education 8% basic education 5% specialist education 5% higher education	50% primary education 40.1% secondary education 6.6%, higher education 3.3% basic education
Religion	Orthodox, Baptist	Orthodox
Language spoken by parents	Romanian (with some dialect words), Ukrainian	Romanian

**Table 3:** Use of wild plants for food by Romanians on both sides of the border (Romanian #, Russian @, Ukrainian $, and * Hutsul dialect used by interviewees to name plants).

Latin Name, Family/Voucher Specimen	Local Name	Parts Used	Preparation	UA R	R R
*Achillea millefolium* L. Asteraceae, NB070, NB117	Tиcячeлиcтник @ (Tysiachelystnyk), Coada-șoarecului #, дepeвий $ бeлий @ (derevyi belyi) Coada-șoricelului #	Aerial parts	Tea	9	
*Arctium lappa* L., Asteraceae, NB149	Лопyx $ (Lopukh)	Roots	Coffee	1	
*Arctostaphylos uva-ursi* (L.) Ericaceae	Urșana #	Fruits	Fresh snack	2	
*Armoracia rusticana* P.Gaertn., B.Mey. and Scherb. Brassicaceae, NB028, NB212 SB031	Hrean #, xpин @ (Khryn), xpiн $ (Khrin)	Roots	Seasoning (beetroots)	8	
			Souse	1	
			Salad with meat		1
			Mustard		2
			Fresh snack as a starter	2	1
			Salad with beetroots	2	14
			For pickled cucumbers	2	
		Leaves	For fermented cucumbers	3	
			Murătură	1	4
			Sarmale		1
*Arnica montana* L. Asteraceae	Arnica #	Flowers	Tea	2	
*Artemisia absinthium* L. Asteraceae NB051	Pelin #	Aerial parts	Tea	1	
*Artemisia dracunculus* L. Asteraceae SB015, SB029	Tarhon #	Aerial parts	Borshch		1
*Atriplex hortensis* L. Amaranthaceae NB088 SB004, SB018	Lobodă #	Aerial parts	Borshch		1
			Sarmale		10
			Soup		1
*Betula* spp. Betulaceae NB041, NB115	Бepeзa $ (bereza), Mesteacăn #	Sap	Alcoholic drink	1	
		Sap	Drink	4	
			Fermented for winter	1	
		Leaves	Tea	1	
*Carpinus betulus* L. Betulaceae	Carpen #	Wood	Smoked meat	1	
*Carum carvi* L. Apiaceae NB037 SB007	Săcărică #, Chimion #	Aerial parts	Tea	3	
		Seeds	Rachiu		2
			Seasoning	1	2
*Crataegus monogyna* Jacq. Rosaceae	Mălăieș #	Fruits	Raw	1	
*Crataegus* spp. Rosaceae NB066	Бояpышник @ (boyarishnik)	Fruits	Tea	2	
*Equicetum* spp. Equisetaceae	Barba ursului #	Roots	Tea	1	
*Fagus sylvatica* L. Fagaceae NB168 SB060	φaг # (fag), fag #	Wood	Smoked meat	5	19
*Fragaria vesca* L., Rosaceae, NB004 SB094	Frăguța # зeмляникa, @ (zemlyanyka), frag #	Fruits	Compote	2	1
			Dessert	2	
			Jam	5	7
			Fresh snack and with cream	1	5
			Snack	1	
			Tea	4	
			Syrup		1
*Humulus lupulus* L. Cannabaceae NB182 SB081	Hamei #	Flowers	Beer		3
*Hypericum* spp. Hypericaceae, NB185	Звipобiй $ (Zvirobii) Звepобой @ (zveroboi), Звipобой @,$ (zviroboj), Pojárniță #	Aerial parts	Tea	9	
*Malus domestica* L. Rosaceae, SB038	Măr #	Wood	Smoked meat	1	2
*Matricaria chamomilla* L. Asteraceae NB164	Pомaшкa $ (Romashka) Romaniț #, Romaniță #	Aerial parts	Tea	14	
*Mentha* spp. Lamiaceae NB080, NB079, NB172	Menta #, Mintă ciuparata # Mintă #	Aerial parts	Tea	6	
*Mentha longifolia* (L.) L. Lamiaceae	Izmă #	Aerial parts	Tea		1
*Plantago major* L. Plantaginaceae NB077	Pătlagină #	Leaves	Salad	1	
		Roots	Tea	1	
*Papaver rhoeas* L. Papaveraceae SB044a, b, c	Mac #	Seeds	Cake		5
*Prunus avium* (L.) L. Rosaceae SB059	Cireș #, чepeшня $ (chereshnia), Cireșe #	Wood	Smoked meat	5	9
*Prunus cerasus* L. Rosaceae SB045	Bишня $ (Vyshnia), Vișine #	Wood	Smoked meat	4	4
*Prunus domestica* L. Rosaceae	Perje #, Perj # Cливa $ (slyva)	Wood	Smoked meat	4	8
*Pyrus pyraster* (L.) Burgsd. Rosaceae	Prăsad #	Fruits	Dried	1	
*Quercus* spp. Fagaceae SB056	Дyб $ (dub), Stejar #	Leaves	Preservative (Murătură)	1	
			For fermented cucumbers	4	1
			For pickled cucumbers and tomatoes	2	
*Robinia pseudoacacia* L. Leguminosae, SB041	Salcâm #	Flowers	Recreational tea		3
			Dessert		1
*Rosa* spp. (including *Rosa canina* L.), Rosaceae NB083	Mălăieș #	Fruits	Juice	2	
			Jam	2	
			Tea	1	
*Rosa* spp. (including *Rosa rugosa*), Rosaceae SB023	Pозa (roza) $, trandafir #	Flowers	Syrup	2	
			Jam	4	10
			Tea		3
*Rubus idaeus* L. Rosaceae SB009, SB071, NB082	Zmiură #, Zmeură # Maлинa $ (malyna)	Leaves	Dried	1	
		Aerial parts	Tea		2
		Fruits	Cake	2	
			Juice	2	4
			Compote	7	14
			Frozen	1	
			Raw	2	2
			Jam	14	15
			Preserve	2	
			Syrup	1	6
			Tea	5	
			Uzvar	1	
*Rubus* spp. (including *Rubus fructicosus* L.) Rosaceae NB001, NB062, NB63 SB073	Чepникa @ (chernyka), Mur #, Mur din padure #, Ojana $ Zmeură neagră #	Fruits	Compote	2	4
			Fresh	7	
			Jam	6	10
			Juice	1	5
			Marmalade with sugar	1	
			Syrup		2
*Rumex acetosa* L. NB081 Polygonaceae	Măcriș-acru #, Măcriș #	Leaves	Soup	3	
			Borshch	4	
*Rumex* spp. Polygonaceae SB076	Шiвa (shiva) $ Stejă # Ștevie #	Leaves	Soup	1	
			Borshch	2	
			Sarmale	2	2
*Sambucus nigra* L. Adoxaceae SB084	Ochio lupului # Soc #	Fruits	Jam	3	
			Socată		7
			Beer		1
			Juice		3
			Tea	1	2
*Symphytum officinale* L. Boraginaceae	Tătăneasă #	Roots	Tea	1	
*Taraxacum campylodes* G.E.Haglund, Asteraceae NB083, SB063	Kyльбaбa $ (kulbaba) Păpădie # Curul găiini #	Roots	Coffee	1	
		Leaves	Salad	5	
			Jam	1	
		Flowers	Tea	1	
			Jam		2
*Syringa vulgaris* L. Oleaceae	Бyзок бiлий $ (busok bilyi)	Flowers	Tea	1	
*Thymus serpyllum* L. Lamiaceae SB001	Cimbrișor #	Aerial parts	Tea	2	1
*Thymus* spp. *Thymus vulgaris* L., Lamiaceae SB090	Cimbrișor #, Cimbru #	Aerial parts	Soup	1	
			Tea	1	1
			Seasoning		11
*Tilia* spp. (including *Tilia cordata* Mill.) Malvaceae, SB017	Tei #, липa $ (lipa)	Flowers	Tea	8	1
*Trifolium* spp., Leguminosae NB076, NB102	Trifoi #	Aerial parts	Tea	3	
*Tussilago farfara* L. Asteraceae NB118 SB065, SB085, NB072	мaти й мaчyxa $ (maty y machukha), podbal #, подбaл # (podbal), mati-maciuha $	Aerial parts	Sarmale	13	8
			Tea	2	
			Galuște		1
			Rolls	2	2
*Urtica dioica* L. Urticaceae SB088, NB152	Kpопивa $ (kropyva), кpaпивa @ (krapiva), Urzica #	Aerial parts	Borshch	4	2
		Young aerial parts	Omelets	1	
		Leaves	Salad	2	
			Scrambled eggs	1	
			Tea	1	
			Soup	15	4
			Stew	2	
			Puree		1
*Vaccinium myrtillus* L. Ericaceae, NB060, SB006	Aφини * (afyny), cernika @, aφыны * (afynu), afină #	Fruits	Jam	3	13
			Preserved with sugar	2	
			Afinătă		2
			Fresh	2	
			Juice		3
			Compote		5
*Vaccinium vitis-idaea* L. Ericaceae, NB061, SB010	Гогдзи * (gogdzy), Merișor #	Fruits	Fresh	2	
			Jam		2
			Tea	1	
*Viburnum opulus* L. Adoxaceae NB157	Kaлинa $ (kalyna), calină #	Fruits	Raw with sugar	2	
			Tea		2
*Vitis vinifera* L. Vitaceae	Vie #	Leaves	Sarmale		2

**Table 4:** Factor of informant consensus. (FIC—factor of informant consensus).

Food Categories	Romania, Number of Uses	Number of Taxa	FIC	Ukraine, Number of Uses	Number of Taxa	FIC
Alcoholic drink	6	4	0.4	1	1	0
Borshch	4	3	0.3333	10	3	0.7778
Cakes	9	3	0.75	4	2	0.6667
Compote	30	5	0.8621	14	3	0.8462
Jam	61	8	0.8833	46	6	0.8889
Juice	15	6	0.6429	11	5	0.6
Fermenting and preserves	4	2	0.6667	19	3	0.8889
Fresh snack	4	2	0.6667	18	8	0.5882
Salad	10	1	1	10	4	0.6667
Sarmale	22	5	0.8095	17	3	0.875
Seasoning	13	2	0.9167	9	2	0.875
Smoking meat	42	3	0.9512	14	4	0.7692
Soup	11	2	0.9	17	3	0.875
Syrup	7	3	0.6667	1	1	0
Tea	10	5	0.5556	79	15	0.8205

**Table 5:** Comparison of Jaccard similarity indexes (JI) for wild food plants used in Bukovina.

Group	Romanians in Ukraine and Romania, JI	Romanians and Hutsuls in Romania, JI	Hutsuls and Romanians in Ukraine, JI	Hutsuls in Ukraine and Romania, JI [6,8]
All food uses	45.65	61.11	52	55/42.5

## Data Availability

The datasets generated for this study are available from the authors upon reasonable request. Data from ERC project DiGe will be fully available after the project ends.

## List of Abbreviations

NWFPs	Non-wood forest products
LEK	Local Ecological Knowledge
DUR	Detailed use report
JI	Jaccard similarity index
RR	Romanians in Romanian Bukovina
UAR	Romanians in Ukrainian Bukovina
UAH	Hutsuls in Ukrainian Bukovina
RH	Hutsuls in Romanian Bukovina
